# Glycolysis in the tumor microenvironment shapes dendritic cell function and antitumor immunity

**DOI:** 10.3389/fimmu.2026.1744671

**Published:** 2026-02-09

**Authors:** Bo Zhang, Linlin Zhao, Huzi Li, Na Wang, Xuerui Wang, Lihan Shang, Bingsheng Sun, Fanming Kong

**Affiliations:** 1 Department of Oncology, First Teaching Hospital of Tianjin University of Traditional Chinese Medicine, Tianjin, China; 2 National Clinical Research Center for Chinese Medicine, Tianjin, China; 3 Tianjin Cancer Institute of Traditional Chinese Medicine, Tianjin, China; 4 Department of Lung Cancer, Tianjin Medical University Cancer Institute & Hospital, National Clinical Research Center for Cancer, Tianjin, China; 5 Tianjin’s Clinical Research Center for Cancer, Tianjin, China; 6 Key Laboratory of Cancer Prevention and Therapy, Tianjin, China

**Keywords:** dendritic cells, glycolysis, immune tolerance, immunotherapy, metabolic reprogramming, tumor microenvironment

## Abstract

Dendritic cells (DCs) are central orchestrators of antitumor immunity, but their functions are markedly curtailed by glycolysis-dominated metabolic constraints in the tumor microenvironment (TME). This review focuses on two interconnected dimensions: tumor-derived metabolic stressors that suppress DC activation and the intrinsic metabolic programs of DC subsets that define their immunogenic potential. Lactate accumulation, hypoxia, adenosine signaling, and lipid overload disrupt antigen cross-presentation, type I interferon (IFN-I) production, and DC migration, collectively biasing DCs toward tolerogenic or checkpoint-high states. At the same time, subset-specific metabolic wiring—such as reliance on oxidative phosphorylation (OXPHOS) and fatty acid oxidation (FAO) in conventional type 1 DCs (cDC1s), glycolysis-dependent Th17-skewing capacity in conventional type 2 DCs (cDC2s), and pronounced hypoxia sensitivity in plasmacytoid DCs—creates distinct vulnerabilities that can be therapeutically exploited. We further summarize emerging strategies to restore DC metabolic fitness, including blockade of tumor glycolysis, intrinsic DC metabolic rewiring, modulation of immunometabolites and redox balance, use of natural products and nanomaterials, and rational combinations with radiotherapy or immune checkpoint blockade. Finally, we outline translational priorities such as single-cell and spatial mapping of DC metabolic heterogeneity, development of metabolism-linked biomarkers, and integration of DC-targeted interventions into existing immunotherapy frameworks. Together, these insights position DC metabolism as a critical lever to reprogram the TME and to enable more durable antitumor immunity.

## 1 Introduction

Dendritic cells (DCs) are professional antigen-presenting cells that play a crucial role in bridging innate and adaptive immunity. By capturing, processing, and presenting antigens, DCs initiate cytotoxic CD8^+^ T cell responses and orchestrate the polarization of CD4^+^ T cells, making them indispensable for antitumor immunity (1–3). However, tumors exploit metabolic and signaling constraints to impair DC function, undermining immune surveillance and leading to suboptimal clinical outcomes (4).

A hallmark of cancer metabolism is aerobic glycolysis, often referred to as the Warburg effect, which sustains malignant proliferation while simultaneously reshaping the tumor microenvironment (TME). Tumor cells consume large amounts of glucose and release lactate, resulting in nutrient depletion and acidosis that suppress immune effector functions (5, 6). Clinically, increased glycolytic activity correlates with immune evasion, reduced DC infiltration, and poorer survival outcomes (6). DCs comprise transcriptionally and functionally distinct subsets, including cross-presenting conventional type 1 dendritic cells (cDC1s), CD4^+^ T cell–priming conventional type 2 dendritic cells (cDC2s), interferon-producing plasmacytoid DCs (pDCs), and inflammatory monocyte-derived DCs (moDCs), each of which can be differentially shaped by tumor metabolic stress (1, 4). In hypoxic and lactate-rich regions of the TME, pDCs adopt tolerogenic states, which are characterized by defective type I interferon (IFN-I) production, further facilitating tumor progression (7, 8).

Metabolic competition provides a second layer of suppression. Tumor cells upregulate glucose transporters 1–3 (GLUT1–3) to monopolize glucose uptake, a feature that is associated with diminished DC abundance and poor prognosis (9). Additional suppressive signals—including oxidized lipids, nitric oxide (NO), adenosine, and endoplasmic reticulum (ER) stress—disrupt DC maturation, cross-presentation, and cytokine release (10–12). This paradox underscores the fact that, despite the abundance of tumor antigens, T cell priming remains insufficient, posing a significant barrier to effective immunity (4).

In addition to these external constraints, DCs also undergo intrinsic metabolic reprogramming. At baseline, DCs rely predominantly on oxidative phosphorylation (OXPHOS) and fatty acid oxidation (FAO) to meet their energy demands (13). Upon activation through Toll-like receptor (TLR) engagement, DCs rapidly switch to glycolysis via key innate immune–related signaling pathways involving TANK-binding kinase 1 (TBK1) and IκB kinase ϵ (IKKϵ)—hereafter referred to as the TBK1–IKKϵ axis—together with mechanistic target of rapamycin complex 1 (mTORC1) and hypoxia-inducible factor 1α (HIF-1α), thereby supporting cytokine production and costimulatory signaling (14, 15). While this glycolytic shift is crucial for acute immune responses, dysregulation of metabolic checkpoints such as pyruvate kinase M2 (PKM2) and 6-phosphofructo-2-kinase/fructose-2,6-bisphosphatase 3 (PFKFB3) can drive dysfunctional phenotypes, marked by impaired antigen presentation and upregulation of programmed death-ligand 1 (PD-L1) (16, 17).

Among DC subsets, cDC1s are the most strongly linked to favorable clinical outcomes through their role in cross-presenting tumor antigens to CD8^+^ T cells. cDC1s predict responsiveness to checkpoint blockade, with Batf3-dependent cDC1s being essential for the efficacy of anti-CD137 and anti-programmed cell death protein 1 (PD-1) therapies (18). Additionally, CD4^+^ T cell-mediated licensing through CD40–major histocompatibility complex class II (MHC-II) interactions further supports the process of cross-priming (19). These findings underscore that the metabolic fitness of cDC1s is a critical factor for sustaining durable T cell immunity.

Building on these insights, this review is organized around three interrelated conceptual axes. First, we explore tumor-derived metabolic constraints, such as glycolysis-driven acidosis, hypoxia, and lipid peroxidation, which profoundly impair DC differentiation and function within the TME. Second, we examine intrinsic metabolic programs—including glycolysis, fatty acid oxidation (FAO), and oxidative phosphorylation (OXPHOS)—that regulate DC activation, subset specialization, and their ability to orchestrate immune responses. Finally, we discuss therapeutic strategies to reprogram DC metabolism, offering a means to restore their immunogenic activity and enhance the efficacy of current immunotherapies. By understanding how these layers of metabolic regulation intersect, we provide a mechanistic framework for designing precision metabolic interventions that can shift DCs from a tolerogenic to an immunostimulatory state, ultimately amplifying durable antitumor immunity.

## 2 Tumor-derived metabolic constraints on DC function

Tumor cells undergo significant metabolic reprogramming to support uncontrolled proliferation, creating a TME marked by nutrient deprivation, lactate accumulation, hypoxia, and lipid peroxidation. These metabolic stressors converge to impair DC maturation, antigen cross-presentation, and migration, while promoting tolerogenic differentiation (1). Importantly, different DC subsets—cDC1s, cDC2s, and pDCs—exhibit distinct vulnerabilities to these metabolic constraints (20).

### 2.1 Lactate accumulation and acidification

Accelerated tumor glycolysis, driven by lactate dehydrogenase A (LDHA) and exported via monocarboxylate transporters 1/4 (MCT1/4), results in persistent lactate release and extracellular acidosis. This lactate-enriched environment suppresses antigen processing, IFN-I signaling through the stimulator of interferon genes (STING) pathway, and CD8^+^ T cell priming (5, 21). Inhibition of MCT1 restores cross-presentation and reprograms intratumoral DCs toward immunogenic phenotypes (21). Notably, robust DC-derived lactate production is most prominently observed upon acute activation (e.g., TLR ligands such as LPS), exposure to inflammatory cytokines, or hypoxia-driven HIF-1α signaling, whereas quiescent DCs generally maintain lower glycolytic flux (14, 22, 23). Available evidence further suggests that lactate output is not uniform across DC lineages: inflammatory moDCs often display stronger glycolytic engagement upon activation than steady-state cDC subsets, whereas pDCs tend to retain a more oxidative metabolic profile; however, systematic head-to-head comparisons in tumor settings remain limited (13, 22). Furthermore, T cells form localized acidic niches in tumor-draining lymph nodes, further dampening effector function (24). Collectively, these findings support a dual-source lactate model, with contributions from both tumor cells and DCs, as a major barrier to effective antitumor immunity.

### 2.2 Glucose competition and nutrient withdrawal

Tumor cells monopolize glucose uptake by overexpressing GLUT1–3 and hexokinase 2 (HK2), a feature associated with poor prognosis and reduced DC infiltration (6, 9). This metabolic competition deprives DCs of the substrates required for TLR-induced glycolytic activation, impairing cytokine secretion, migration, and survival. Although intrinsic glycogenolysis provides a temporary buffer during early activation, this compensatory mechanism collapses under sustained nutrient stress (15, 25). Restoration of fructose-1,6-bisphosphatase 1 (FBP1) activity has been shown to promote DC maturation and interleukin-33 (IL-33) secretion in lung adenocarcinoma, illustrating therapeutic opportunities to reprogram DC metabolism (26).

### 2.3 Hypoxia, ROS, and ferroptotic stress

Hypoxia stabilizes HIF-1α, reprogramming DCs toward a tolerogenic phenotype. In hepatocellular carcinoma (HCC), the HIF-1α–adenosine axis recruits pDCs with impaired IFN-I competence (8, 23). Hypoxia also induces the production of reactive oxygen species (ROS), which damages mitochondria and triggers lipid peroxidation. Oxidized lipids disrupt peptide–MHC-I assembly (10, 27), while ferroptosis-like stress preferentially eliminates cross-presenting DCs (28). Nanomedicine-based approaches that reduce glycolysis and lipid peroxidation can partially restore antigen presentation (29). In pancreatic ductal adenocarcinoma (PDAC), excessive hypoxia-driven glycolysis results in particularly profound DC dysfunction, linking metabolic stress directly to immune exclusion (30).

### 2.4 Adenosine and glycan-mediated tolerance

Extracellular adenosine triphosphate (ATP) hydrolysis by CD39/CD73 generates adenosine, which signals via A2A receptors to suppress IL-12 secretion, DC trafficking, and cross-priming (31). Hypoxia amplifies this pathway by upregulating ectonucleotidases (8). Simultaneously, tumor-associated glycosaminoglycans (GAGs) promote regulatory T cell (Treg) expansion and skew DCs toward a tolerogenic state (32). This highlights that immunoregulation in the TME is driven not only by metabolites but also by structural features such as glycans.

### 2.5 Tumor-derived lipid cues

Polyunsaturated fatty acid (FA)–bound α-fetoprotein and other tumor lipids reprogram DCs by reducing OXPHOS and promoting FA synthesis (33). These lipid signals impair antigen processing and reinforce tolerogenic programming.

### 2.6 Integration and implications

**Figure 1:**
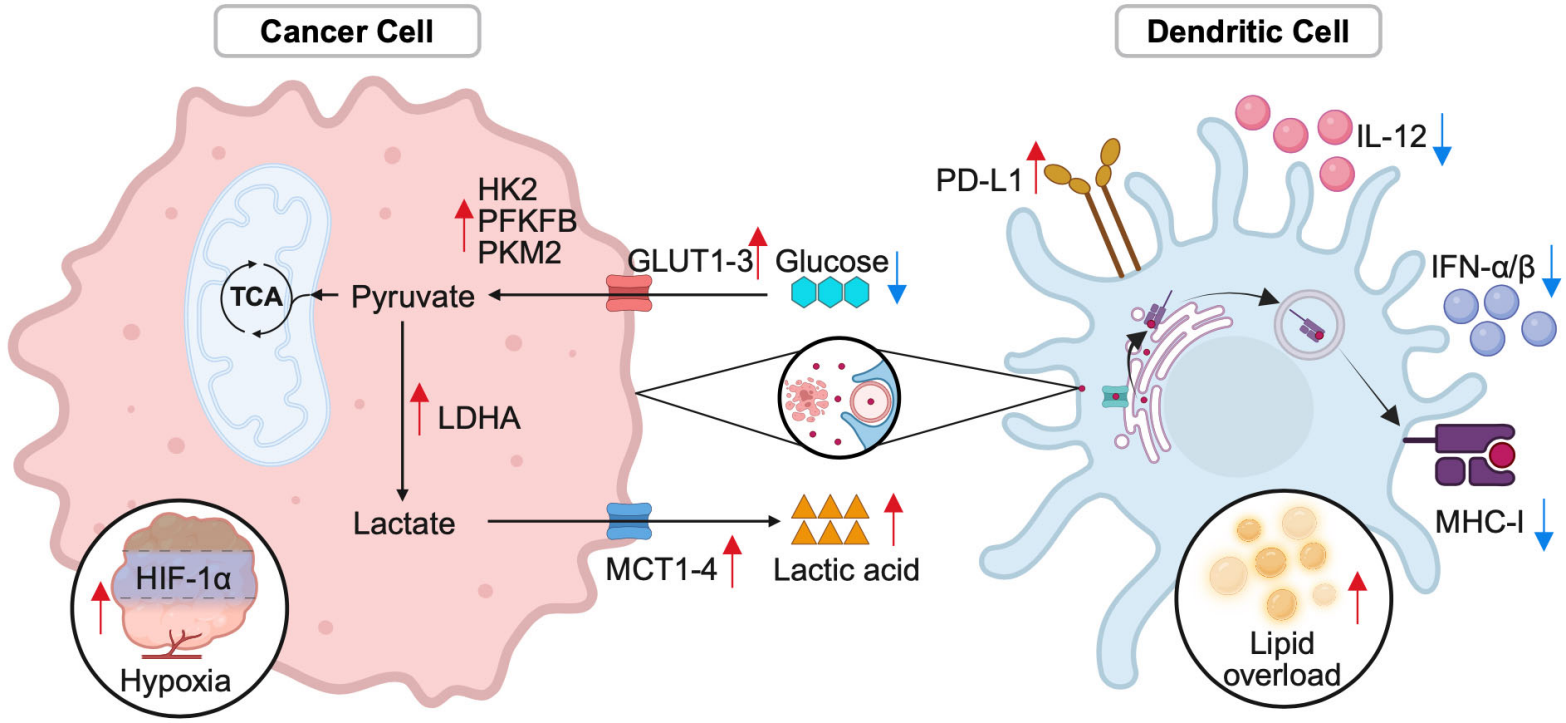
Tumor-derived metabolic stressors impair DC antigen presentation. In the TME, tumor-derived metabolic stressors, including enhanced glycolysis and nutrient competition, severely impair DC function. Elevated glucose uptake through GLUT1–3 and lactate export via LDHA/MCT1–4 contribute to nutrient depletion and acidification, which disrupt antigen processing and cross-presentation. Key glycolytic enzymes, such as PKM2, not only promote IL-12 secretion but also induce PD-L1 expression, creating an immune-suppressive environment. Overexpression of HK2 correlates with reduced immune infiltration, while FBP1 restoration supports DC maturation and IL-33 release. Additionally, PFKFB isoforms fine-tune glycolytic flux in DCs. Tumor-induced hypoxia activates HIF-1α signaling, and lipid overload further hinder peptide–MHC-I loading and type I interferon secretion. Collectively, these metabolic and environmental stressors reprogram DCs, pushing them toward an immunosuppressive state, which weakens T cell priming and facilitates immune evasion.

Tumor-derived immunosuppression operates through multiple, convergent metabolic axes that collectively undermine DC function. Lactate accumulation and acidosis disrupt cellular homeostasis and impair antigen processing; glucose competition and glycogen depletion deprive DCs of essential bioenergetic substrates; hypoxia and ROS-induced ferroptosis trigger oxidative damage; elevated adenosine and aberrant glycans promote tolerogenic signaling; and lipid overload drives metabolic drift toward an immunosuppressive phenotype. Together, these stressors converge to disable antigen cross-presentation and T cell priming. Targeting tumor glycolysis through LDHA/MCT1 inhibition, restoring FBP1 activity to balance glycolytic flux, preventing ferroptosis, inhibiting adenosine-mediated signaling, and reprogramming lipid metabolism emerge as promising strategies to restore DC functionality and reinstate durable antitumor immunity. A summary of the principal tumor-derived stressors and their inhibitory effects on DC function is provided in **Figure 1**; **Table 1**.

**Table 1:** Tumor-derived metabolic constraints on DC function.

Tumor-derived factor	Key mediators	Effect on DCs	Functional consequence	Representative tumor types
Excess glycolysis → lactate accumulation	LDHA; MCT1/4	Acidifies the TME; impairs antigen processing and STING signaling	↓ Cross-presentation; ↓ CD8^+^ T-cell priming	Glioma; PDAC
Hypoxia	HIF-1α stabilization	Increases mitochondrial ROS and lipid peroxidation	Ferroptosis-like stress; DC loss	HCC; PDAC
Adenosine signaling	CD39/CD73 → eADO → A2A	Suppresses IL-12 secretion and DC trafficking	↓ T-cell priming	Lung cancer; breast cancer
Lipid overload/oxidized lipids	PUFA; oxidized lipids	Blocks peptide–MHC-I loading	Antigen presentation disabled	Ovarian cancer; melanoma
Gasotransmitters	NO; CO	Inhibit respiration and lysosome–ER fusion → defective endosome–ER routing	Impaired cross-presentation	TME-wide

Symbols: “→” indicates causal effect; “↓” decreased function.

## 3 Tumor cell glucose metabolism constrains DC antigen presentation

Building upon the tumor-derived metabolic pressures discussed previously, tumor glycolysis directly impairs DC antigen presentation and cross-priming. Glycolytic enzymes, transporters, and metabolites act as critical regulatory checkpoints, linking tumor metabolism to immune dysfunction.

### 3.1 Lactate efflux and LDHA/MCT1 dependency

Tumors with elevated LDHA expression and MCT1/4-mediated lactate export sustain continuous lactate efflux, resulting in persistent acidosis in the TME. This lactate-rich environment impairs endosomal trafficking, peptide–MHC-I loading, and antigen escape from the cytosol (21, 27). Inhibition of LDHA or blockade of MCT1 restores cross-presentation in cDC1s and enhances CD8^+^ T cell priming, particularly when combined with checkpoint inhibitors (5, 18, 21). In glioma models, MCT1 inhibition with 3-bromopyruvate alleviates lactate-induced dysfunction (34). These findings establish lactate export as a key translational checkpoint linking tumor glycolysis to DC paralysis.

### 3.2 Glycolytic enzymes as immune checkpoints

Certain glycolytic enzymes function as dual regulators of DC immunogenicity. For example, PKM2 promotes IL-12 secretion but simultaneously induces PD-L1 expression, skewing DCs toward a tolerogenic state (16, 17). HK2, frequently overexpressed in renal carcinoma, correlates with diminished immune infiltration and poor survival (6). Conversely, FBP1 antagonizes glycolysis, restores DC maturation, and promotes IL-33 secretion (26). The roles of different PFKFB isoforms also vary depending on context: PFKFB4 drives glycolysis and pentose phosphate pathway activity in colon cancer, whereas PFKFB2 supports glycolytic bursts during DC activation (35, 36). These enzymes not only regulate bioenergetics but also act as metabolic checkpoints determining immune tolerance versus activation.

### 3.3 Glucose transporters and glycogen buffering

Tumor cells monopolize glucose by overexpressing GLUT1–3, depriving DCs of the glucose required for TLR-induced glycolytic activation (9). This nutrient gating impairs cytokine release, survival, and migration of DCs. Excessive basal glycolysis in monocytes also inhibits their differentiation into migratory DCs (37). In contrast, DCs rely on intrinsic glycogenolysis as a temporary buffer during early activation, supporting IL-1β secretion and cross-priming (25). Restoring glucose access via GLUT1 blockade or nanovesicle-based metabolic interventions rescues DC function (38).

### 3.4 ROS and ferroptosis under glycolytic stress

High glycolytic flux in the TME elevates ROS and lipid peroxidation, which further exacerbates DC dysfunction. Oxidized lipids disrupt peptide–MHC-I assembly, while ferroptosis-like stress selectively eliminates cDC1s responsible for cross-presentation (10, 27, 28). This apparent selectivity is likely multifactorial, reflecting both cell-intrinsic susceptibility to lipid peroxidation during antigen processing and microenvironmental conditions that favor lipid oxidation and ROS accumulation (e.g., hypoxia, impaired redox buffering, and lipid overload) in metabolically stressed TMEs (39). Mechanistically, cross-presentation is tightly coupled to endosomal and ER membrane remodeling and lipid handling, processes that increase exposure to peroxidizable lipids and oxidative stress. In this context, cDC1-like cross-presenting programs may become disproportionately vulnerable when GPX4-dependent lipid detoxification capacity is exceeded or when mitochondrial ROS rises during sustained antigen processing, providing a plausible mechanistic basis for the observed enrichment of ferroptosis-like loss within cross-presenting DC states. Nanoparticle-based strategies that mitigate ferroptosis or reprogram lipid metabolism can restore DC cross-presentation (29). Recent studies further highlight ferroptosis as a context-dependent immunometabolic program that can reshape antitumor immunity and antigen-presenting cell function in metabolically constrained TMEs (40). These findings highlight how glycolysis-driven oxidative cascades disable antigen presentation and contribute to immune suppression in the TME.

### 3.5 Adjuvants and metabolic reprogramming

Beyond tumor inhibition, certain adjuvants directly reprogram DC metabolism. Carbomer-based adjuvants, for instance, help establish a favorable OXPHOS- ROS balance, which promotes antigen escape and peptide loading (41). Similarly, monophosphoryl lipid A (MPLA)-adjuvanted immunotherapies fine-tune glycolysis while preserving CD8^+^ T cell priming (42). These examples underscore that the rational design of adjuvants can reshape hostile TMEs into environments conducive to effective cross-priming and antitumor immunity.

### 3.6 Integration and implications

Tumor glycolysis imposes multifaceted constraints on DC antigen presentation through a series of convergent mechanisms. Excessive lactate efflux driven by LDHA and MCT1 impairs DC trafficking and cross-presentation, thereby weakening T cell priming. Dysregulation of the PKM2/PD-L1 and HK2/FBP1 metabolic axes establishes checkpoints that determine the balance between immune tolerance and activation. Furthermore, GLUT monopolization and glycogen depletion in the TME deprive DCs of essential nutrients and disrupt their intrinsic energy buffering capacity. The accumulation of ROS and ferroptosis exacerbates lipid peroxidation, contributing to the selective loss of cDC1s. Conversely, administration of immunometabolic adjuvants can restore cross-presentation and reinvigorate antitumor T cell responses. Clinically, therapeutic targeting of these metabolic pathways holds the potential to synergize with checkpoint blockade and DC-based vaccines, offering a promising avenue to overcome tumor-induced immunosuppression.

## 4 Intrinsic glucose metabolism programs DC function

Beyond the suppression imposed by tumors, DCs rely on intrinsic metabolic programs that regulate antigen presentation, cytokine production, and migration. These metabolic networks integrate glycolysis, glycogenolysis, serine biosynthesis, FAO, autophagy, and redox balance, ultimately determining whether DCs adopt immunogenic or tolerogenic phenotypes.

### 4.1 Inflammasome–metabolism coupling

The adaptor protein apoptosis-associated speck-like protein containing a CARD (ASC), well-known for its role in inflammasome assembly, also regulates mitochondrial dynamics and glycolytic thresholds. During infection, ASC-dependent regulation of mitochondrial ROS determines whether DCs undergo immunogenic activation or metabolic collapse (43). In tumor models, ASC deficiency disrupts cross-presentation and CD8^+^ T cell priming, positioning inflammasome–metabolism coupling as an early checkpoint in DC activation.

### 4.2 mTOR–epigenetic cross-talk

mTORC1 activation drives glycolysis and anabolic metabolism, sustaining costimulatory molecule expression and cytokine release (44). Tuberous sclerosis complex 1 (TSC1), a negative regulator of mTORC1, maintains metabolic–epigenetic balance and CD8^+^ T cell homeostasis (45). Loss of this regulatory restraint accelerates glycolytic exhaustion and tolerance, demonstrating how nutrient sensing integrates with chromatin remodeling to shape DC fate.

### 4.3 Glycolytic enzymes as dual checkpoints

Branchpoint glycolytic enzymes act as dual regulators of DC immunogenicity. PKM2 enhances IL-12 secretion but simultaneously induces PD-L1 expression, driving immune tolerance (16, 17). PFKFB3 sustains glycolytic flux but is overactivated in tumor-associated DCs, driving dysfunction (15). Long noncoding RNAs such as MIR4435-2HG amplify mTORC1-driven glycolysis, impairing antigen presentation (46). These findings underscore the role of glycolytic enzymes as pivotal checkpoints linking metabolic regulation to immune outcomes.

### 4.4 Glycogen metabolism in early activation

Glycogen serves as a rapid energy reserve during TLR and C-type lectin receptor (CLR) stimulation. Mobilized glycogen fuels glycolysis and nicotinamide adenine dinucleotide phosphate (NADPH) production, sustaining inflammasome activation and cytokine secretion (15). Loss of glycogenolysis compromises IL-1β release and cross-priming, highlighting its importance for early metabolic flexibility (47).

### 4.5 Ligand-specific metabolic codes

Receptor–ligand interactions impose unique metabolic “codes” that govern DC function. Engagement of α2–3 sialic acids with Siglecs suppresses glycolysis and promotes tolerance, while β-glucan ligation via CLR–Syk signaling induces glycolysis and ROS production independently of TLRs (48, 49). These interactions allow DCs to integrate environmental cues and tailor their metabolic responses accordingly.

### 4.6 Serine biosynthesis and IFN programs

Diversion of glucose into serine biosynthesis via phosphoglycerate dehydrogenase (PHGDH), phosphoserine aminotransferase 1 (PSAT1), and phosphoserine phosphatase (PSPH) supports nucleotide synthesis and redox balance, reinforcing antiviral and antitumor responses (50). In pDCs, unfolded protein response (UPR) signaling channels glucose into one-carbon metabolism (51). IFN-I signaling further remodels metabolism, with TLR-induced IFN-I responses requiring PI3K–mTOR–p70S6K signaling (52, 53).

### 4.7 Viral infection models as parallels

Viral infection models highlight the metabolic flexibility of DCs. Respiratory viruses activate poly (ADP-ribose) polymerases 1 (PARP1), depleting NAD^+^ and impairing mitochondrial respiration (54). Inhibition of glycolysis blocks retinoic acid-inducible gene I (RIG-I)–mediated antiviral signaling, while influenza infection dynamically reprograms glycolysis and OXPHOS to sustain effector functions (55, 56). These parallels illustrate that intact glycolysis–OXPHOS coupling is indispensable for DC immunity.

### 4.8 Environmental and dietary triggers

Extrinsic factors, including diet and environmental cues, also influence DC metabolism. High fructose exposure drives glycolysis and lipogenesis in human DCs, exaggerating proinflammatory outputs (57). Similarly, allergen fusion proteins activating TLR5 enhance glycolysis and FA synthesis (58). These findings underscore the role of diet and adjuvants as external modulators of DC activity.

### 4.9 Crosstalk with tissue niches

Stromal and epithelial metabolism further imprints DC phenotypes. In tuberculosis, alveolar epithelial cells induce HIF-1α–nitric oxide synthase 2 (NOS2) signaling in DCs, skewing glycolysis toward NO production (59). Toxoplasma gondii infection reprograms DC glucose and lipid fluxes (60). Similarly, tumor stroma may impose similar metabolic imprints on infiltrating DCs, suggesting a hierarchical regulation of DC metabolism by tissue niches.

### 4.10 Balancing pro- and anti-inflammatory outputs

Peroxisome proliferator-activated receptor gamma coactivator 1-beta (PGC-1β) sustains OXPHOS and restrains inflammatory gene expression (61). Conversely, interferon-gamma (IFN-γ) induces a Warburg-like glycolytic shift, driving proinflammatory outputs but predisposing DCs to exhaustion (62). These dual outcomes emphasize the need for therapeutic “safety windows” in metabolic interventions.

### 4.11 ROS, Nrf2, and tolerogenic stabilization

ROS accumulation skews DCs toward tolerance. Nrf2 activation enhances OXPHOS and FAO while restraining glycolysis, stabilizing tolerance (63). Inhibition of Nrf2 restores immunogenicity, confirming ROS adaptation as a central determinant of functional polarity.

### 4.12 Autophagy–glycolysis coupling

Autophagy intersects with glycolytic regulation to modulate DC metabolism. Deficiency in autophagy-related protein 5 (ATG5) induces hyper-glycolysis and mitochondrial stress, enhancing antigen presentation but accelerating DC exhaustion (64). This highlights the need for balance between acute glycolytic surges and long-term metabolic fitness in DCs.

### 4.13 OXPHOS and FAO balance

Mitochondrial respiration and FAO are essential for DC viability and cross-presentation. Excessive FAO via carnitine palmitoyltransferase 1a (CPT1a)/PPARα signaling drives semimature DC phenotypes, whereas balanced FAO/OXPHOS maintains durable antigen presentation (65–67).

### 4.14 Integration and implications

Intrinsic metabolic programs equip DCs with remarkable plasticity, enabling them to adapt to a wide range of microenvironmental cues. Several molecular checkpoints orchestrate this metabolic–immune interface, including ASC–inflammasome coupling, the TSC1–mTOR regulatory balance, PKM2/PD-L1 signaling, glycogen reserves maintenance, and Nrf2-driven antioxidant tolerance. Among these processes, antigen cross-presentation is particularly vulnerable, as factors such as ER stress, UPR activation, lipid overload, ferroptosis, mitochondrial dysfunction, and dysregulated gasotransmitter signaling impair peptide–MHC-I assembly. Therefore, selective modulation of glycolytic enzymes, serine biosynthesis, and redox regulators—while preserving physiological cues from tissue and pathogens—represents a rational approach to reinvigorate DC function and restore antitumor immunity.

## 5 Cross-presentation under metabolic stress

Cross-presentation is one of the most metabolically demanding DC functions, requiring coordinated antigen uptake, endosomal trafficking, ER-dependent peptide loading, and mitochondrial ATP/ROS support. In the TME, chronic stressors—including lipid overload, ER stress, defective organelle quality control, and altered death modalities—severely impair this pathway, compromising CD8^+^ T cell priming.

### 5.1 ER stress, UPR, and inflammatory noise

Persistent ER stress activates the inositol-requiring enzyme 1 alpha (IRE1α)–X-box binding protein 1 (XBP1) axis, which drives lipogenesis and abnormal lipid droplet accumulation (11, 68, 69). High XBP1 activity correlates with defective cross-presentation and poor prognosis (70, 71). UPR signaling also elevates IL-23, skewing DCs toward T helper 17 cells (Th17) polarization—creating a paradox where cross-priming is impaired, but inflammatory “background noise” is heightened (72, 73). Endocrine cues further reinforce this: vitamin D3 derivatives redirect lipid metabolism toward FA synthesis, stabilizing tolerance and further limiting antigen presentation (74–77).

### 5.2 Lipid overload and ferroptotic stress

Tumor-associated DCs often accumulate triglycerides and cholesterol esters, impairing proteasome-dependent processing (78, 79). Hypoxia-driven ROS generate oxidized lipids that disrupt peptide-loading complexes (10, 27). Ferroptosis-like lipid peroxidation selectively eliminates cross-presenting cDC1s (28). Antioxidants or ferroptosis inhibitors can partially restore function (80). Natural metabolites also modulate outcomes: spermidine activates forkhead box O3 (FOXO3) to counter inflammatory dysfunction, whereas Protosappanin A biases DCs toward tolerance (81, 82). These findings illustrate the fine balance between immunogenic and tolerogenic rewiring in the TME.

### 5.3 Cell death modalities and antigen salvage

The mode of tumor cell death influences antigen salvage. Pyroptotic corpses expose F-actin “crowns” that engage C-type lectin domain family 9 member A (CLEC9A)/dendritic cell natural killer lectin group receptor-1 (DNGR-1), facilitating efficient antigen uptake (83). In contrast, ER stress and lipid peroxidation impair endosomal routing, limiting antigen recovery. Thus, tumor-driven cell death modalities act as “hardware thresholds,” constraining cross-presentation efficiency.

### 5.4 Mitochondrial quality control and autophagy

Mitochondrial integrity is indispensable for cross-presentation. Defective mitophagy causes ROS overload and impaired ER trafficking (84, 85). Enhancing autophagy restores antigen presentation, improves vaccine potency, and prolongs DC survival (86, 87). Conversely, blocking acetyl-CoA carboxylase-1/2 drives FAO dependence and semimature states with reduced priming (88). Organelle quality control thus emerges as a key determinant of DC competence.

### 5.5 Organelle crosstalk and gasotransmitters

Mitochondria–ER–endosome networks coordinate antigen routing. NO suppresses respiration and endosomal maturation (89). Carbon monoxide (CO), via heme oxygenase-1, disrupts ATP supply and lysosomal fusion (90). Environmental triggers also reshape thresholds: TLR5-ligand fusion proteins boost glycolysis/lipogenesis, and high fructose exposure exacerbates proinflammatory responses in human DCs (57, 58).

### 5.6 Tumor-derived cascades and systemic spillover

Cross-presentation is also influenced by upstream signals. In tuberculosis, alveolar epithelial cells activate HIF-1α–NOS2 signaling in DCs, diverting glycolysis toward NO production (59). Toxoplasma gondii infection reprograms DC glucose and lipid fluxes (60). Similarly, tumor stroma may impose similar pre-programming on infiltrating DCs, constraining their antigen-presenting capacity.

### 5.7 Metabolic nanomedicine to rescue cross-presentation

Nanotechnology provides strategies to counter multifactorial suppression in DCs. Biomimetic nanocarriers co-delivering LDHA inhibitors and cholesterol esterification blockers restore lipid balance and normalize lactate metabolism (29). Metal–phenolic networks simultaneously regulate glycolysis and lipid oxidation (91). Other strategies include STING agonist–loaded nanovesicles with GLUT1 blockade and lncRNA-targeted modulation of HIF-1α–C-C chemokine receptor type 7 (CCR7) circuits to restore DC migration (38, 92). These approaches integrate metabolic rewiring with antigen delivery, offering clinically actionable outcomes.

### 5.8 Summary and implications

DC cross-presentation is profoundly impaired by multiple tumor-induced stressors that disrupt ER and mitochondrial homeostasis. ER stress and activation of the UPR compromise antigen processing and peptide loading. Concurrently, vitamin D–induced lipogenesis, excessive lipid accumulation and oxidation, and defective mitochondrial quality control collectively exacerbate metabolic dysfunction and antigen presentation failure. Additional insults such as maladaptive cell death, and tumor-derived gasotransmitters such as NO and CO, further distort DC immunogenicity. Moreover, environmental inputs—notably dietary fructose and microbial ligands—can recalibrate the activation threshold of DCs within the TME.

Therapeutically, interventions such as UPR inhibition, antioxidant therapy, ferroptosis modulation, immunometabolite supplementation, and nanoplatform-based delivery systems have shown promise in restoring cross-presentation and reinvigorating DC-mediated antitumor immunity.

## 6 Subset-specific metabolic wiring of DCs

High-dimensional profiling has shown that DC subsets are endowed with distinct metabolic programs that can be traced back to the progenitor stage and are later reinforced by tissue-specific cues (93–95). Transcriptional signatures within common DC progenitors already bias cells toward glycolysis-dominant or OXPHOS-dominant fates, and these are further shaped by local nutrient and oxygen availability (96). This “metabolic hardwiring” helps explain why individual subsets respond differently to tumor-derived stress and highlights the need for subset-tailored metabolic interventions.

### 6.1 cDC1: cross-presentation under mitochondrial and lipid stress

cDC1s excel at antigen cross-presentation and depend on intact mitochondrial respiration with a calibrated level of glycolysis to sustain proteasome-dependent antigen routing and CCR7-mediated migration. Their lineage is driven by Batf3/interferon regulatory factor 8 (IRF8) and CD103^+^ progenitors (94, 97, 98). In breast cancer, T-cell immunoglobulin and mucin domain 3 (TIM-3) signaling modulates cDC1 responsiveness to chemotherapy, and subset-specific adaptation to the TME has been confirmed (20, 99). Metabolically, excess lipid uptake and peroxidation compromise antigen processing, whereas FAO preserves mitochondrial fitness and antigen presentation (100, 101). Clinically, Batf3-dependent cDC1s are indispensable for anti-CD137/PD-1 efficacy, and *in situ* mobilization of cDC1s can overcome resistance to anti–PD-L1 therapy (18, 102).

Therapeutic lever: reinforcing FAO/OXPHOS while limiting lipid peroxidation may sustain cDC1 persistence and cross-priming in hostile TMEs.

### 6.2 cDC2 and inflammatory DCs: glycolysis-driven Th17 bias

cDC2s specialize in CD4^+^ T-cell priming and IL-23/Th17-type responses. Upon PRR stimulation they undergo a robust glycolytic upshift, including PFKFB2-driven glycolysis (36). Human CD1c^+^ DCs link glycolytic signatures to CD8^+^CD103^+^ T cell priming (103–105). When glycolysis becomes excessive, CCR7-dependent migration is restricted and tolerance is favored (37). The glucose–mTORC1–HIF-1α axis further limits helper T-cell support, while NCoR1 fine-tunes glycolysis–FAO balance (106, 107). Inflammatory DCs (inf-DCs) display an even more pronounced glycolytic program and high IL-23 output: co-stimulation with anti-IgE and Pam3CSK4 promotes Th17 skewing, and human inf-DCs can directly drive Th17 differentiation through glycolytic licensing (108, 109).

Therapeutic lever: partial dampening of glycolysis or selective targeting of the IL-23/Th17 axis may recalibrate cDC2/inf-DCs toward productive immunity without extinguishing their priming capacity.

### 6.3 pDCs: oxidative metabolism for IFN-I

pDCs rely on OXPHOS and FAO to sustain IFN-I production (110, 111). AMP-activated protein kinase (AMPK) preserves mitochondrial remodeling and IFN-α secretion, while a certain level of glycolysis remains necessary for antiviral signaling (47, 112). In lactate-rich TMEs, pDCs undergo epigenetic rewiring toward tolerance and lose IFN-I competence (7). ER stress diverts glucose into one-carbon metabolism, and mitochondrial DNA oxidation drives aberrant T follicular helper (TFH) support (51, 113).

Therapeutic lever: AMPK agonists and antioxidants that stabilize mitochondria may restore IFN-I programs in pDCs under metabolic stress.

### 6.4 LAMP3^+^ migratory DCs: metabolic brakes on trafficking

LAMP3^+^ DCs, defined by single-cell and spatial transcriptomics, form a migratory bridge between tumors and draining lymph nodes (114). They frequently display ER-stress and lipid-metabolism signatures and tend to adopt checkpoint-high, tolerogenic states. NF-κB–dependent steady-state signaling constrains their activation, whereas the lncRNA Dpf3 suppresses HIF-1α–driven glycolysis and limits CCR7-dependent migration (92, 115).

Therapeutic lever: targeting HIF-1α–glycolysis checkpoints or rebalancing ER/lipid homeostasis may unlock the immunogenic potential of LAMP3^+^ DCs.

### 6.5 moDCs: vaccine optimization and metabolic fragility

moDCs, widely used in vaccine platforms, display metabolic profiles that predict survival and immunogenicity (116). Serum-free granulocyte–monocyte progenitor (GMP) media help stabilize these states, but tumor-derived or exogenous lactate can drive tolerogenic drift (22, 117). In contrast, α-ketoglutarate restores redox metabolism, whereas metformin may promote FOXO3a-linked tolerance (118, 119). Additional regulators include EGF-like repeats and discoidin I-like domains 3 (EDIL3)–AMPK signaling suppressing glycolysis and progesterone conditioning that skews metabolism toward tolerance (120, 121).

Therapeutic lever: supplementation with redox cofactors (e.g. α-ketoglutarate), selective “glycolytic brakes,” or hormone-based modulators may improve moDC vaccine efficacy while limiting tolerogenic reprogramming.

### 6.6 Integration and perspective

**Figure 2:**
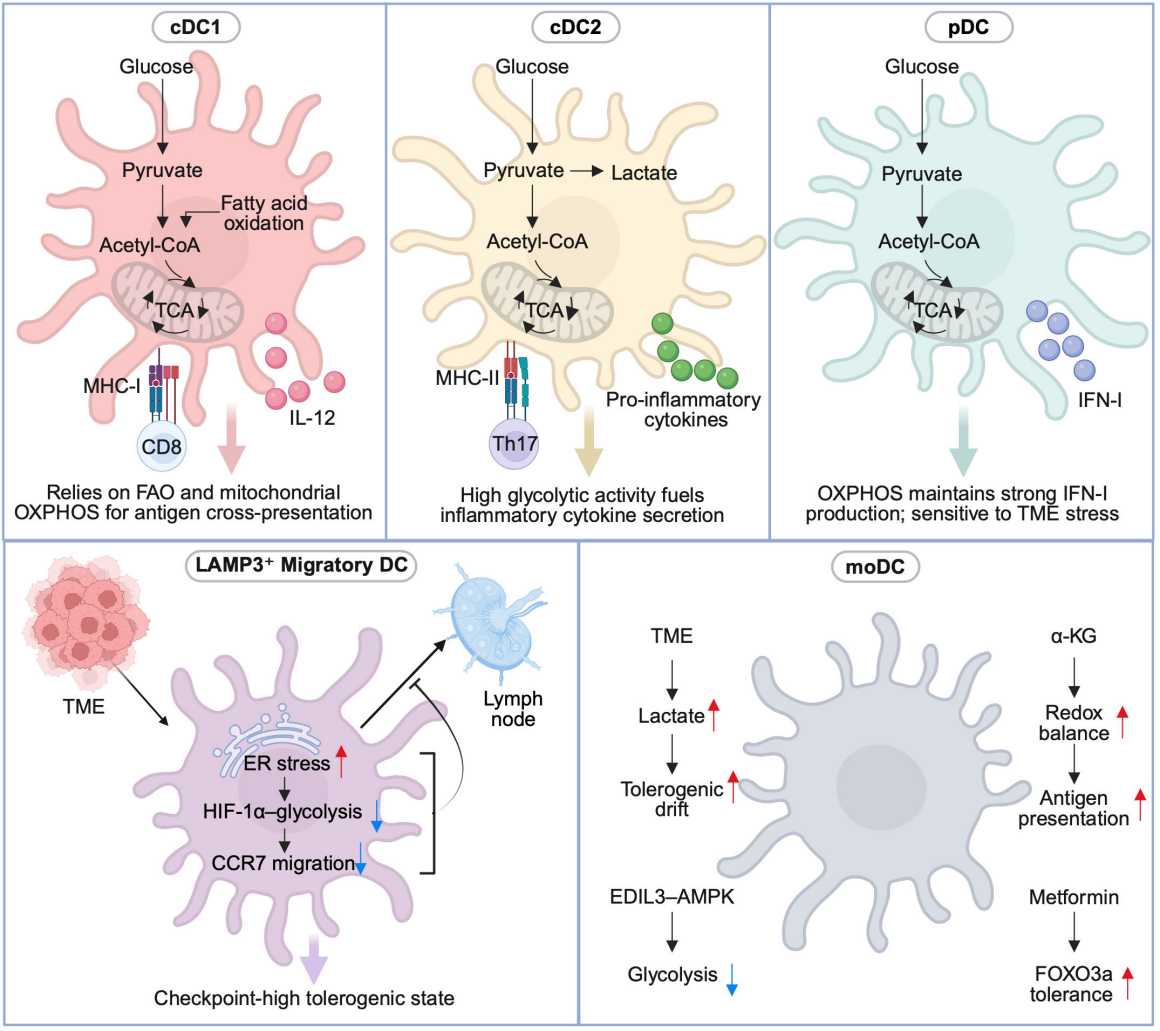
Subset-specific metabolic wiring of DCs. Distinct DC subsets exhibit unique metabolic programs aligned with their immune functions and vulnerabilities. cDC1 relies on balanced glycolysis and FAO/OXPHOS to sustain cross-presentation under mitochondrial and lipid stress; supporting FAO/OXPHOS while limiting lipid peroxidation enhances persistence in hostile TMEs. cDC2 and inflammatory DCs are glycolysis-driven, promoting IL-23/Th17 polarization; partial modulation of glycolysis or IL-23 signaling restores productive immunity. pDCs depend on FAO and OXPHOS for IFN-I production, sustained by AMPK and redox homeostasis; antioxidants and AMPK activators restore antiviral competence under lactate stress. LAMP3^+^ migratory DCs bridge tumors and lymph nodes but are metabolically restrained by ER stress and HIF-1α–glycolysis checkpoints; restoring ER/lipid balance reactivates CCR7-dependent migration. moDCs exhibit metabolic fragility in vaccine settings; supplementation with α-ketoglutarate and selective glycolytic brakes can preserve immunogenicity.

Subset-specific metabolic wiring demonstrates how lineage origin, tissue imprinting, and environmental stress jointly shape DC behavior in the TME. Programs ranging from glycolysis-dominant cDC2s/inf-DCs to OXPHOS-reliant pDCs and migration-restrained LAMP3^+^ DCs reveal both metabolic vulnerabilities and therapeutic entry points. These insights provide a rationale for precision immunometabolic interventions. In practice, strategies that (i) enhance FAO/OXPHOS in cDC1s, (ii) modulate glycolysis and Th17 polarization in cDC2s/inf-DCs, (iii) preserve mitochondrial integrity in pDCs, and (iv) relieve HIF-1α–mediated migratory brakes in LAMP3^+^ DCs together outline a roadmap toward next-generation DC-targeted immunotherapy. Metabolic wiring and therapeutic entry points for major DC subsets are summarized in **Figure 2**; **Table 2**.

**Table 2:** Subset-specific metabolic programs and vulnerabilities of dendritic cells.

DC subset	Dominant metabolic program	Key vulnerabilities	Functional consequence	Potential therapeutic levers
cDC1	OXPHOS, FAO	Lipid peroxidation; ferroptosis; mitochondrial stress	Impaired cross-presentation; reduced CCR7-guided migration	Reinforce FAO/OXPHOS; block lipid ROS/peroxidation
cDC2	Glycolysis (PFKFB-driven bursts)	Excess basal glycolysis → tolerogenic reprogramming; migration restraint	Th17 skewing; weaker lymph-node trafficking	Partial dampening of glycolysis; IL-23/Th17-axis tuning
pDC	OXPHOS, FAO	Lactate- and hypoxia-induced ER stress	Reduced type I IFN production; blunted antiviral/tumor surveillance	AMPK agonists; antioxidants; mitochondrial stabilizers
LAMP3^+^ DC	HIF-1α–driven glycolytic brakes with ER/lipid signatures	CCR7-pathway restraint; checkpoint-high/tolerogenic state	Poor lymph-node trafficking; tolerogenic bias	Target HIF-1α; rebalance ER/lipid homeostasis; restore CCR7
moDC	Mixed (glycolysis + OXPHOS)	Lactate-induced drift; hormone or serum conditioning	Vaccine-phenotype instability; tolerance skewing	α-KG and redox support; serum-free media; mild glycolytic brakes

Symbols: “→” indicates causal effect.

## 7 Therapeutic strategies to rewire DC metabolism – opportunities and challenges

Because metabolism is a major determinant of DC fate, it offers multiple entry points for therapeutic intervention. Current approaches to restoring DC fitness in the TME can broadly be categorized into extrinsic strategies that relieve tumor-imposed metabolic stress and intrinsic strategies that reprogram DC bioenergetics.

### 7.1 Reducing tumor glycolytic pressure

Excessive tumor glycolysis promotes lactate accumulation and acidosis, both of which suppress DC cross-presentation and CD8^+^ T-cell priming. Lowering this glycolytic pressure can partially restore DC function. Biomimetic nanocarriers co-delivering an LDHA inhibitor and a cholesterol esterification blocker have been shown to re-establish antigen presentation and cytotoxic T lymphocyte activation (29). Pharmacologic inhibition of MCT1 reprograms cDC1s, cDC2s, and pDCs toward immunogenic phenotypes (21). Comparative metabolomics has further revealed distinct nutrient partitioning between tumors and myeloid cells, supporting selective “glucose restriction” strategies that starve tumor cells while preserving DC metabolic fitness (122).

### 7.2 Direct reprogramming of intrinsic DC metabolism

DC activity is also shaped by cell-intrinsic metabolic checkpoints. Epigenetic regulators such as PRMT5 control the balance between glycolysis and OXPHOS and thereby influence cytokine secretion (85). Lymphocyte-activation gene 3 (LAG-3) signaling can rewire glycolysis and antigen presentation, while loss of β2-integrin enhances IL-12 production through metabolic reprogramming (123, 124). G protein–coupled receptor 120 (GPR120) signaling suppresses HK2-dependent glycolysis and generates regulatory DCs (125). Notably, this effect appears ligand-dependent (most commonly reported for long-chain fatty acids, including ω-3–derived ligands such as DHA/EPA) and has primarily been demonstrated under defined inflammatory contexts; whether GPR120-mediated metabolic programming occurs uniformly across distinct DC subsets remains to be fully determined. In parallel, cytotoxic T-lymphocyte-associated protein 4 (CTLA-4) blockade destabilizes Tregs in glycolysis-low tumors and synergizes with DC activation (126). Together, these findings indicate that tuning signaling, epigenetic, and adhesion-related checkpoints offers a route to fine-tune DC immunogenicity.

### 7.3 Immunometabolites and redox modulators

Small-molecule immunometabolites provide a softer means of stabilizing DC metabolism. Ethyl pyruvate reduces ROS and supports mitochondrial function (127). Allithiamine redirects glucose toward OXPHOS, limiting lactate production (128). 2-deoxyglucose (2-DG) shows dose- and context-dependent effects: at low doses it can favor tolerance, whereas higher doses during antigen exposure may suppress immunity (80, 129). Itaconate reduces IL-23 secretion under acute inflammatory conditions, thereby limiting excessive DC-driven Th17 responses, but in chronic stress or tumor-associated contexts it can also induce PD-L1 expression through mtDNA–STING signaling, potentially reinforcing immunosuppressive programs. These context-dependent effects underscore the importance of timing, metabolic state, and microenvironmental cues in determining whether itaconate exerts immunoregulatory or immunosuppressive outcomes (130, 131). Operationally, the balance likely depends on (i) the timing of itaconate exposure relative to DC priming, (ii) whether mitochondrial damage and mtDNA release are present, and (iii) the magnitude and duration of STING activation, which together shape whether anti-inflammatory cytokine restraint or checkpoint reinforcement predominates. Overall, these agents highlight that redox and carbon-flux control can reinforce DC fitness if used under well-defined conditions.

### 7.4 Natural compounds as DC modulators

Plant- and steroid-derived compounds represent versatile immunometabolic modulators that can be integrated with vaccines or checkpoint therapy. Emerging evidence indicates that many natural compounds exert their immunomodulatory effects by rewiring cellular metabolism, thereby shaping dendritic cell differentiation, antigen processing, and tolerogenic versus immunogenic fate decisions (132, 133). Ginsenoside Rg5 mobilizes glycogen and enhances efferocytosis (134). Apigenin exerts both antiproliferative and immunomodulatory effects (135). Vitamin D3 derivatives promote fatty acid synthesis, stabilizing tolerogenic programs in DCs (74–77). Other agents, such as the arylmethylaminosteroid SC1O and kinsenoside, modulate PI3K–AKT–FoxO1 signaling (136, 137). Targeting solute carrier family 7 member 11 (SLC7A11) can further improve antigen quality control (138). Collectively, these compounds provide bioactive scaffolds for restoring DC metabolic competence and illustrate how natural-product–based interventions can be leveraged to fine-tune DC metabolism within immunosuppressive microenvironments (132).

### 7.5 Biomaterial-based strategies integrating metabolism and antigen co-delivery

Recent advances in biomaterials have enabled simultaneous control of antigen delivery, DC metabolism, and the TME. Glioblastoma-associated myosin (gMSN) nanoparticles act as epigenetic nano-adjuvants that enhance mucosal vaccine efficacy (139). Trojan-yeast–based systems deplete intratumoral glucose, thereby restoring DC immunogenicity and promoting effective T-cell activation (140). Moreover, nanovesicles and metal–drug coordination networks have been designed to co-deliver GLUT1 inhibitors together with STING agonists, synchronizing metabolic suppression with innate immune activation (38). Intranasal hybrid vesicles and hypoxia-mimicking hydrogels facilitate DC recruitment and antigen trafficking under low-oxygen conditions (141–144). Additional platforms—such as immune scaffolds leveraging CTRP9–SLC7A11 signaling to refine apoptotic antigen quality, or carbomer-based adjuvants that induce metabolically favorable states for cross-presentation—illustrate how biomaterials can serve as integrative metabolic–antigen tools (41, 145). Collectively, these approaches establish a framework for next-generation DC-centered immunotherapies.

Collectively, these emerging platforms exemplify an integrated biomaterial–metabolism–antigen framework, establishing a conceptual foundation for next-generation dendritic cell–centered immunotherapies.

### 7.6 Radiotherapy and checkpoint blockade

**Figure 3:**
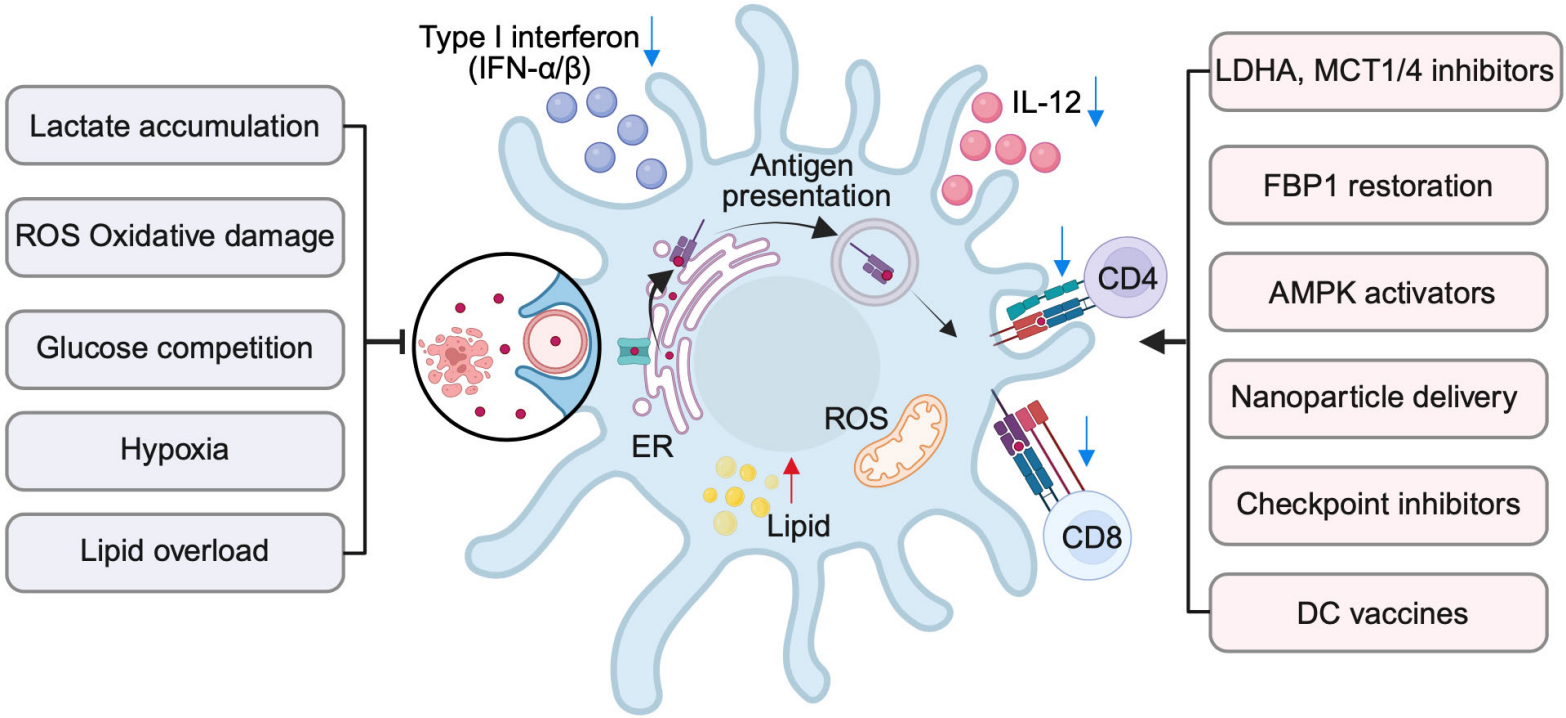
Metabolic stress suppresses DC cross-presentation and therapeutic strategies restore function. Tumor-derived stressors—including lactate accumulation, glucose competition, hypoxia, lipid overload, and ROS—disrupt ER homeostasis, peptide loading, and antigen routing in DCs. These changes impair cross-presentation and reduce cytokine secretion, such as IL-12 and type I interferons, thereby weakening CD8^+^ T cell priming and limiting CD4^+^ T cell support. Targeted interventions can restore DC function: inhibition of LDHA/MCT1/4, restoration of FBP1 activity, and AMPK activation rebalance cellular metabolism; nanomedicine-based delivery enhances antigen processing; and immunotherapy approaches, including checkpoint blockade and DC vaccines, potentiate T cell activation. Together, these strategies highlight metabolic reprogramming as a promising lever to overcome tumor-induced suppression and enable effective antitumor immunity.

Metabolic interventions can also be combined with existing cancer therapies. Radiotherapy generates ROS that can impair DCs, but this can be mitigated by nanomedicine-based antioxidants (146). Ultrasound-triggered metabolic inhibitors enhance sonodynamic immunotherapy, and microwave ablation combined with glycolysis inhibition promotes central memory CD8^+^ T-cell differentiation (147, 148). Clinically, DC vaccines combined with dasatinib have elicited responses in checkpoint-refractory melanoma (149). At the checkpoint interface, Batf3-dependent cDC1s remain indispensable for anti-CD137/PD-1 efficacy, while PKM2–PD-L1 coupling links glycolysis to immune escape (17, 18, 150). These data support the rationale for pairing DC metabolic rewiring with immunotherapy to achieve more durable responses (**Figure 3**).

### 7.7 Integration and perspective

Therapeutic targeting of DC metabolism constitutes a multilayered framework that integrates extrinsic, intrinsic, and combinatorial strategies to restore immunogenic function. Extrinsic modulation seeks to relieve tumor-derived glycolytic stress—typically via LDHA or MCT1 inhibition—to reduce lactate, improve oxygenation, and create space for DC activation. Intrinsic rewiring focuses on regulators such as PRMT5, LAG-3, β2-integrin, and lipid checkpoints to optimize antigen presentation and cytokine output. Immunometabolites and redox stabilizers (e.g. ethyl pyruvate, allithiamine, itaconate) add a tunable layer that can support mitochondrial integrity without fully overhauling DC metabolism. Natural compounds offer additional, clinically tractable tools. Biomaterial-based platforms unify these efforts by co-delivering antigens and metabolic modulators, and combination with radiotherapy or checkpoint blockade broadens their translational scope. Taken together, these interventions converge on a common goal: restoring DC cross-presentation, improving vaccine efficacy, and sustaining durable antitumor immunity.

## 8 Translational insights and clinical evidence

### 8.1 From bench to bedside: why DC metabolic fitness matters

The variable efficacy of DC-based vaccines in the clinic suggests that metabolic fitness is a decisive determinant of therapeutic success. In melanoma vaccine cohorts, immunometabolic readouts—such as extracellular acidification rate (ECAR), oxygen consumption rate (OCR), and GLUT expression—correlated strongly with overall survival, indicating their potential value as biomarkers for quality control and patient stratification (116). Refinements in vaccine manufacturing, including the use of serum-free and chemically defined media, helped stabilize moDC metabolic states and improved batch-to-batch reproducibility (117). Additional translational gains have come from combination approaches: vascular antigen–targeted DC vaccines combined with dasatinib induced durable responses in checkpoint-refractory melanoma, while ultrasound-triggered metabolic suppression platforms enhanced DC recruitment and cross-presentation, amplifying ongoing immunotherapy (147, 149). Together, these data argue that embedding metabolic endpoints into DC-vaccine design can increase clinical predictability.

However, several practical barriers currently limit the routine incorporation of these metabolic assays into clinical trial pipelines. ECAR and OCR measurements typically require freshly isolated, viable cells and specialized platforms, which can be difficult to harmonize across centers and are often incompatible with archived clinical specimens. Similarly, expression-based biomarkers (e.g., GLUT, PGK1, and TUBA1C) are sensitive to tissue processing, intratumoral heterogeneity, and dynamic immune states, complicating cross-cohort comparability and threshold definition. In addition, assay cost, technical expertise requirements, and regulatory validation represent non-trivial constraints for large-scale implementation. Therefore, clinical translation will likely require standardized operating procedures, surrogate readouts compatible with fixed tissue or liquid biopsy samples, and integration into adaptive trial designs that support longitudinal immune–metabolic monitoring.

### 8.2 Lessons beyond cancer: DC–T cell revival

Findings from infectious and chronic viral disease models highlight the interdependence of DC and T-cell metabolic competence. In patients with human immunodeficiency virus (HIV), ex vivo DC therapy restored CD8^+^ T-cell responses only when both DCs and T cells retained mitochondrial function. Exhausted PD-1^+^/TIGIT^+^ T cells with impaired respiration required a combination of metabolic augmentation and checkpoint blockade to recover (151). Similarly, IFN-I signaling remodels DC metabolism to sustain antiviral activity, whereas glycolysis inhibition directly blocks RIG-I–mediated antiviral signaling (53, 55). Influenza infection further illustrates the principle that dynamic switching between glycolysis and OXPHOS is necessary to maintain effector programs (56). These observations can be generalized to cancer: durable benefit is more likely when metabolic fitness is restored in both the antigen-presenting compartment and the responding T cells.

### 8.3 Which DC subsets matter in patients?

Clinical and spatial studies indicate that not all DC subsets contribute equally to therapeutic outcomes. cDC1s are critical for cross-presentation and for responsiveness to immune checkpoint blockade (19). Tumor-derived retinoic acid and liver X receptor (LXR) activation can suppress CCR7-dependent DC migration and thereby attenuate antitumor immunity (152, 153). Conversely, reprogramming cDC2-like programs can drive protective CD4^+^ T-cell immunity in tumors (154). Tissue-resolved profiling further shows that DCs acquire site-specific metabolic and inflammatory imprints: in synovial tissue, myeloid DC subsets diverge in inflammatory versus tolerogenic contexts; in solid tumors, large-scale spatial immunophenotyping has revealed immune–metabolic niches that predict PD-1 outcomes in triple-negative breast cancer (155, 156). Biomarker studies support this view: phosphoglycerate kinase 1 (PGK1) and tubulin alpha-1C chain (TUBA1C) correlate with DC infiltration and survival in lung adenocarcinoma, while GLUT transporter expression stratifies prognosis in head and neck cancers (9, 157, 158). These findings support clinical integration of spatial and metabolic profiling to guide patient selection.

### 8.4 Translational pipelines: where DC metabolism meets modern immunotherapy

Emerging therapeutic pipelines increasingly incorporate metabolic signatures as part of DC-vaccine readouts. Antigen-specific CD8^+^ T cells primed by DC vaccines display metabolic profiles distinct from those induced by peptide vaccines and show improved persistence when combined with PD-1 blockade (159). This supports the inclusion of metabolic biomarkers in release criteria for next-generation DC products.

In parallel, advances in biomaterials extend the translational reach of metabolic modulation: metal–phenolic networks can reprogram both tumor and DC metabolism; metabolite-releasing polymers prolong antigen presentation; and antigen–adjuvant nanovesicles enhance cross-priming in otherwise suppressive TMEs (38, 91, 160). Systemic metabolic context also needs to be considered: adipose tissue from esophageal adenocarcinoma patients displayed therapy-induced metabolic remodeling, suggesting that host metabolism can shape responses to DC-based strategies. Reviews of cancer vaccine trials converge on a similar point: integrating metabolic checkpoints into translational pipelines is likely to improve durability and consistency of clinical benefit (161, 162).

### 8.5 Challenges

Despite substantial progress, several obstacles still limit the clinical translation of DC-focused metabolic interventions. First, tumor heterogeneity produces highly context-specific patterns of immunometabolic suppression, which means that “one-size-fits-all” approaches are unlikely to succeed (143). Second, systemically administered metabolism-targeting agents may cause off-target toxicities, underscoring the need for DC-directed, localized, or biomaterial-based delivery systems to improve specificity and safety (38). Importantly, systemic targeting of central metabolic nodes such as LDHA, GLUT1, or glycolysis with agents including 2-deoxyglucose inherently carries a risk of unintended immune suppression, given the shared metabolic requirements of T cells, macrophages, and host tissues. To mitigate these liabilities, several strategies may be considered, including localized or tumor-restricted delivery when feasible, dose and schedule optimization to exploit therapeutic windows, transient or context-dependent metabolic modulation, and immune-context–guided patient stratification to avoid broadly dampening protective immunity. Where feasible, ex vivo metabolic tuning during DC-vaccine manufacturing may offer a safer translational route than systemic *in vivo* inhibition, because exposure can be tightly controlled and release criteria can be coupled to functional potency assays. Targeted delivery platforms—such as nanocarriers and biomaterials—may further improve DC-focused metabolic rewiring by enhancing tissue specificity and reducing systemic exposure; however, these systems introduce additional translational risks that must be explicitly addressed (38). Key concerns include biodistribution to the liver and spleen, complement activation, unintended innate immune stimulation, and material-dependent biopersistence or long-term immunotoxicity. Accordingly, rigorous *in vivo* profiling of pharmacokinetics, biodistribution, and immunotoxicity, alongside validation of DC-targeting specificity and on-treatment immune monitoring, should be incorporated early in development. From a regulatory perspective, these platforms also require robust CMC control (batch-to-batch reproducibility, material characterization, and endotoxin/sterility assurance) and, for long-lived materials, longer-term safety follow-up to exclude delayed immunotoxicity. Third, from a translational standpoint, standardized biomarker integration is still lacking: measurements of ECAR and OCR, expression of metabolic effectors such as GLUT, PGK1, and TUBA1C, and spatial profiling of DC–T-cell niches should be embedded into trial design to enable adaptive stratification (9, 157, 158). Looking forward, several priorities can guide clinical development. Metabolic biomarkers should be validated—ideally via liquid biopsy–based surrogates—to report DC and T-cell fitness in real time. cDC1 licensing should be formalized as a prerequisite for achieving durable synergy between DC-based approaches and immune checkpoint blockade (ICB) (18, 19). Subset coverage should be broadened to include cDC2s and migratory DC populations so as to reinforce CD4^+^ T-cell–mediated immunity (154). Finally, systemic metabolic profiling—such as incorporating adipose- or serum-derived signatures—should be used to stratify patients and predict responsiveness to immunometabolic therapies (161).

Together, these steps can help move DC metabolic modulation from a mainly mechanistic field to a clinically actionable pillar of next-generation cancer immunotherapy.

## 9 Conclusion & perspectives

### 9.1 DC metabolism as a lever for antitumor immunity

Across preclinical models and early clinical studies, DCs emerge as central integrators of metabolic context and adaptive immune output. Tumor-derived pressures—including glycolytic overload, lactate accumulation, hypoxia, adenosine signaling, and lipid stress—converge to impair DC maturation, cross-presentation, and IFN-I production. At the same time, DC-intrinsic bioenergetics—namely the balance between glycolysis, OXPHOS, and FAO—determines whether DCs sustain antitumor immunity or drift toward tolerogenic states (1, 4). These two layers imply that effective restoration of DC function will require a dual approach: alleviating extrinsic metabolic stress in the TME and, in parallel, recalibrating DC-intrinsic metabolic programs (5, 15).

### 9.2 The double-edged role of glycolysis

Glycolysis occupies a paradoxical position. On the tumor side, blocking glycolysis—especially lactate production and export—relieves metabolic suppression and permits DC cross-priming to resume (5, 21). On the DC side, however, glycolysis and short glycogen-fueled bursts are indispensable for pattern-recognition receptor (PRR) signaling, cytokine release, and migration (15). Broad, non-selective glycolytic inhibition therefore risks weakening DC vaccines or adjuvant responses. A pragmatic solution is to prioritize tumor-directed interventions (e.g. LDHA or MCT1 inhibition) while simultaneously supporting DC metabolism, guided by immunometabolic biomarkers derived from both tumor and immune compartments (9, 116).

### 9.3 Subset-tailored interventions

Functional and metabolic heterogeneity among DC subsets argues for precision modulation rather than uniform escalation. cDC1s profit from enhanced mitochondrial respiration and FAO, which sustain cross-presentation and CD8^+^ T-cell priming; these effects can be reinforced through CD4^+^ T-cell–mediated licensing (19). In contrast, cDC2s depend more heavily on glycolytic flux to support IL-6 and IL-23 production and thus favor Th17 polarization (36, 109, 161). pDCs are predominantly oxidative and require intact mitochondrial pathways to maintain IFN-I secretion, but they become epigenetically and metabolically silenced in lactate- or hypoxia-rich TMEs (7, 112). Meanwhile, LAMP3^+^ migratory DCs, meanwhile, experience HIF-1α–linked glycolytic constraints that limit CCR7-dependent lymph node trafficking (92, 115).

Taken together, these distinctions support the design of “subset-aware” adjuvants that pair antigen delivery with tailored metabolic cues to maximize antitumor immunity.

### 9.4 From mechanism to modality

DC metabolism intersects directly with immune checkpoint pathways, creating opportunities for combinatorial therapy. The PKM2/HIF-1α axis can drive PD-L1 expression on DCs, providing a mechanistic rationale for combining DC-targeted metabolic rewiring with PD-1/PD-L1 blockade or costimulatory agonists (17). In parallel, nanoplatforms that co-deliver antigens and metabolic modulators have been shown to enhance DC persistence, cross-priming, and overall vaccine performance (29, 144). MCT1 inhibition further reestablishes immunogenic DC phenotypes and counters tumor-induced tolerance (21).

Beyond pharmacology, host metabolic state also matters: high-fructose exposure augments glycolytic flux and amplifies proinflammatory DC activation, whereas dyslipidemia perturbs IFN-γ–driven responses and weakens host defense (57, 163).

These observations suggest that nutritional or lifestyle interventions could serve as adjuncts to metabolic or checkpoint-based therapies, although rigorous clinical validation is still needed (164).

### 9.5 Outlook: mapping, measuring, and matching

Moving DC metabolic modulation from mechanism to clinic will require coordinated progress in three areas. First, mapping efforts should employ single-cell and spatial technologies to chart DC heterogeneity and metabolic states across tumor types and treatment contexts (165, 166). Second, measuring needs to focus on scalable biomarkers—ECAR/OCR assays, GLUT and PGK1 expression, and liquid biopsy–based surrogates—to enable real-time monitoring of DC and T-cell fitness in patients (9, 116, 157). Third, matching should integrate DC vaccines, checkpoint blockade, metabolic modulators, and even dietary interventions according to subset-specific vulnerabilities, such as Batf3-dependent cDC1 activity or pDC mitochondrial dependence (19, 112).

If these elements are aligned, DC metabolism can shift from a largely mechanistic topic to a clinically actionable pillar of next-generation immuno-oncology.

## Glossary

2-DG: 2-deoxyglucose
AMP: adenosine monophosphate
AMPK: AMP-activated protein kinase
ASC: apoptosis-associated speck-like protein containing a CARD
ATG5: autophagy-related protein 5
ATP: adenosine triphosphate
CCR7: C-C chemokine receptor type 7
cDC1s: conventional type 1 dendritic cells
cDC2s: conventional type 2 dendritic cells
CLEC9A: C-type lectin domain family 9 member A
CLR: C-type lectin receptor
CMC: chemistry, manufacturing, and controls
CO: carbon monoxide
CPT1a: carnitine palmitoyltransferase 1a
CTL: cytotoxic T lymphocyte
CTLA-4: cytotoxic T-lymphocyte-associated protein 4
CTRP9: C1q/tumor necrosis factor-related protein 9
DCs: dendritic cells
DNGR-1: dendritic cell natural killer lectin group receptor-1
ECAR: extracellular acidification rate
EDIL3: EGF-like repeats and discoidin I-like domains 3
ER: endoplasmic reticulum
FA: fatty acid
FAO: fatty acid oxidation
FOXO3: forkhead box O3
gMSN: glioblastoma-associated myosin
GC: gastric cancer
GLUT: glucose transporter
GLUT1: glucose transporter 1
GLUT1–3: glucose transporters 1–3
GMP: granulocyte-monocyte progenitor
GPR120: G protein-coupled receptor 120
HIF-1α: hypoxia-inducible factor 1α
HIV: human immunodeficiency virus
ICB: immune checkpoint blockade
IFN-I: type I interferon
IFN-γ: interferon-gamma
IKKϵ: IκB kinase ϵ
IL-10: interleukin-10
IL-12: interleukin-12
IL-23: interleukin-23
IL-33: interleukin-33
IRE1α: inositol-requiring enzyme 1 alpha
IRF8: interferon regulatory factor 8
ITIM: immunoreceptor tyrosine-based inhibitory motif
LAG-3: lymphocyte-activation gene 3
LAMP3^+^ DCs: lysosomal-associated membrane glycoprotein 3^+^ dendritic cells
LDHA: lactate dehydrogenase A
LPS: lipopolysaccharide
LXR: liver X receptor
MCT: monocarboxylate transporter
MCT1: monocarboxylate transporter 1
MCT4: monocarboxylate transporter 4
MCT1/4: monocarboxylate transporters 1/4
MHC: major histocompatibility complex
MHC-I: major histocompatibility complex class I
MHC-II: major histocompatibility complex class II
moDCs: monocyte-derived dendritic cells
MPLA: monophosphoryl lipid A
mTORC1: mechanistic target of rapamycin complex 1
mtDNA: mitochondrial DNA
NADPH: nicotinamide adenine dinucleotide phosphate
NK: natural killer
NO: nitric oxide
NOS2: nitric oxide synthase 2
OCR: oxygen consumption rate
OXPHOS: oxidative phosphorylation
PARP1: poly(ADP-ribose) polymerase 1
PD-1: programmed cell death protein 1
PD-L1: programmed death-ligand 1
PDAC: pancreatic ductal adenocarcinoma
PGC-1β: peroxisome proliferator-activated receptor gamma coactivator 1-beta
PGK1: phosphoglycerate kinase 1
pDCs: plasmacytoid dendritic cells
PFKFB3: 6-phosphofructo-2-kinase/fructose-2,6-bisphosphatase 3
PHGDH: phosphoglycerate dehydrogenase
PKM2: pyruvate kinase M2
PPARα: peroxisome proliferator-activated receptor alpha
PRR: pattern recognition receptor
PSAT1: phosphoserine aminotransferase 1
PSPH: phosphoserine phosphatase
RIG-I: retinoic acid-inducible gene I
ROS: reactive oxygen species
SLC: solute carrier
STING: stimulator of interferon genes
TBK1: TANK-binding kinase 1
TBK1–IKKϵ: TANK-binding kinase 1–IκB kinase epsilon axis
TFH: T follicular helper
Th17: T helper 17 cells
TIGIT: T cell immunoreceptor with Ig and ITIM domains
TIM-3: T-cell immunoglobulin and mucin domain 3
TLR: Toll-like receptor
TME: tumor microenvironment
TSC1: tuberous sclerosis complex 1
TUBA1C: tubulin alpha-1C chain
Treg: regulatory T cell
UPR: unfolded protein response
XBP1: X-box binding protein 1
